# Factors Associated with Stunting among Pre-school Children in Southern Highlands of Tanzania

**DOI:** 10.1093/tropej/fmw024

**Published:** 2016-04-27

**Authors:** Chiara Altare, Tefera Darge Delbiso, George Mutembei Mutwiri, Regine Kopplow, Debarati Guha-Sapir

**Affiliations:** ^1^Centre for Research on the Epidemiology of Disasters, Catholic University Louvain, Clos Chapelle-aux-Champs 30, Bte B1.30.15, Brussels 1200, Belgium; ^2^Action Contre La Faim, Research and Analysis Unit, 14/16 Boulevard De Douaumont, Paris 75854, France; ^3^Concern Worldwide Tanzania, 169 Regent Estate, Mikocheni a, Dar Es Salaam, Tanzania; ^4^Concern Worldwide, 52-55 Camden Street, Dublin 2, Ireland

**Keywords:** stunting, growth, maternal support, child care, diet diversity, Tanzania.

## Abstract

**Background:** Stunting is a major public health problem in Africa and is associated with poor child survival and development. We investigate factors associated to child stunting in three Tanzanian regions.

**Methods:** A cross-sectional two-stage cluster sampling survey was conducted among children aged 6-59 months. The sample included 1360 children aged 6-23 months and 1904 children aged 24-59 months. Descriptive statistics and binary and multivariate logistic regression analyses were used.

**Results:** Our main results are: in the younger group, stunting was associated with male sex (adjusted odds ratio [AOR]: 2.17; confidence interval [CI]: 1.52-3.09), maternal absence (AOR: 1.93; CI: 1.21-3.07) and household diet diversity (AOR: 0.61; CI: 0.41-0.92). Among older children, stunting was associated with male sex (AOR: 1.28; CI: 1.00-1.64), age of 4 and 5 (AOR: 0.71; CI: 0.54-0.95; AOR: 0.60; CI: 0.44-0.83), access to improved water source (AOR: 0.70; CI: 0.52-0.93) and to a functioning water station (AOR: 0.63; CI: 0.40–0.98) and mother breastfeeding (AOR: 1.97; CI: 1.18-3.29).

**Conclusions:** Interventions that increase household wealth and improve water and sanitation conditions should be implemented to reduce stunting. Family planning activities and programmes supporting mothers during pregnancy and lactation can positively affect both newborns and older siblings.

## INTRODUCTION

Linear growth failure is a major public health problem in Africa, where more than one-third of the children under 5 are too short for their age [1, 2]. Extensive research has shown the health, economic and intergenerational consequences of stunting: higher risk of dying [3]; poorer psychomotor and mental development and school achievement [4, 5]; loss of human capital and economic productivity in adulthood [6, 7]; in creased risk of chronic diseases [8]; and reduced maternal reproductive outcomes [9].

Stunting often begins *in utero*, as maternal nutrition is the first determinant of the child nutritional status [1, 10], and continues generally during the first two years after birth [11, 12]. Although the pathogenesis of stunting is not yet well-understood, studies have shown that inadequate nutrient intake, infections, unsafe water and poor child care are among its main determinants [13, 14]. Other factors in developing countries include maternal education, socioeconomic status, residence and poor access to health services [15–18].

There is increasing international recognition that efforts to prevent stunting can improve short- and long-term outcomes, at individual, community and country levels [7, 19]. This is reflected in the number of governments joining the Scaling-Up Nutrition (SUN) movement (http://scalingupnutrition.org), and in the inclusion of nutrition-related goals in the World Health Assembly targets, Millennium Development Goals and Sustainable Development Goals.

In Tanzania, a SUN member, 35% of the children under 5 were stunted in 2014 [20], down from 42% in 2010 [21]. In central and southern highlands zones, however, the prevalence of chronic malnutrition reaches 50%. Here, the Government of Tanzania, UNICEF and Concern Worldwide, an international non-governmental organization, are implementing the ‘Bringing nutrition actions to scale in Iringa, Njombe and Mbeya regions of Tanzania’ project. It aims to reduce the prevalence of stunting by 10 percentage points over 5 years, through interventions targeting women and children, as well as strengthening capacities of local authorities. At the beginning of the project, baseline information was collected on infant and young child feeding (IYCF) practices, child and maternal nutritional and health status and household socioeconomic situation.

By analysing these baseline survey data, this paper investigates factors associated with stunting in regions with high stunting prevalence. Differently from the majority of studies from Tanzania, our analysis does not focus on HIV subjects, but on otherwise healthy children without confirmed co-morbidities.

## METHODS

### Study area and population

The study was conducted in the regions of Iringa, Njombe and Mbeya, where 4.4 million people live, 72% of whom live in rural areas [22]. Although these regions receive the highest rainfall and are Tanzania’s bread baskets, stunting prevalence was 51.3%, 51.5% and 36.0%, respectively, in 2014 [20]. These are the second and the third highest values in Tanzania, well above the national prevalence (34.7%). The study population includes children under 5 in rural and urban households in the three regions.

### Study and sampling design

One cross-sectional survey was conducted in each region in November 2013, using a two-stage cluster sampling design. Sixty-three clusters were selected in each region by probability proportional to the size using ENA delta software [23]. Twenty households were chosen in each cluster by random sampling, using a random number table. A complete list of households with children under 5 in each cluster was prepared before the survey date. Households were visited for verification if necessary. Sample size was calculated to detect a 10 percentage point reduction in stunting among children 24–47 months by the end of the project in each region. Power was set at 80%, level of confidence at 95% (one-tailed test), design effect at 1.5 and non-response at 10%. A sample of 501 children in the age group 24–47 months per region was required. A total of 1253 households with children under 5 were targeted in each region to achieve the required sample size.

### Measurements

Data were collected using a standardized questionnaire on a digital data gathering (DDG) device, via face-to-face interview with the main caregiver of the child. The following data were collected for anthropometric measurements of all children under 5: sex, age, weight, height and presence of bilateral pitting oedema. Length was taken for children under 24 months in horizontal position; height was taken standing for older children; both to the nearest 0.1 cm with a standard 130-cm height/length board. Weight was measured with an electronic scale to the nearest 0.1 kg. Stunting was defined as height-for-age *z*-score (HAZ) below −2 SD from the median height of the WHO reference population.

Additional data were collected to reflect selected immediate, underlying and basic causes of undernutrition as illustrated in the UNICEF Conceptual Framework [24]. These were regrouped in child characteristics: IYCF practices, occurrence of diseases, supplementation and treatments received; maternal characteristic: nutritional status, pregnancy and breastfeeding status, workload, habits and supplementation during pregnancy and nutritional information received; and household characteristics: water source, sanitation facilities, use of iodized salt, household dietary diversity and farm diversity. When more than one child aged 6–23 months was present in a sampled household, only data from the youngest child were collected. Definition and measurements of variables used in the analysis are presented in Table 1.

**Table 1.** Definitions and measurement of variables used in the analysis

Variable name	Definition and measurement	Values
*Child level*		
Minimum diet diversity (MDD) [43]	Proportion of children aged 6 to 23 months eating from four or more food groups.	0 if < 4 groups
		1 if ≥ 4 groups
Minimum meal frequency (MMF) [25]	Proportion of children receiving solid, semi-solid or soft food (but also including milk feed for non-breastfed children), the minimum number of times according to their age and breastfeeding status. Minimum is defined as: two times for breastfed infants aged 6 to 8 months; three times for breastfed children aged 9 to 23 months; four times for non-breastfed children aged 6 to 23 months.	0 if MMF not reached;
		1 if MMF reached
Minimum adequate diet (MAD) [25]	Proportion of children aged 6 to 23 months who achieve both MMF and MDD. It is calculated separately for breastfed and non-breastfed children. For breastfed children, it corresponds to the children who reach both MDD and MMF. For non-breastfed children, MAD is based on a six-food-group MDD and requires also two separate milk feeds.	0 if MAD not reached;
		1 if MAD reached
Occurrence of disease	The occurrence of diarrhoea, fever or cough in the two weeks before the survey. Based on caregiver’s recall (children 6 to 23 months of age).	0 if no disease episode; 1 if at least one episode of any of the three conditions
Child vitamin A supplementation	Proportion of children 6 to 23 months of age who received a Vitamin A dose (capsule) in the 6 months previous to the survey. Based on caregiver’s recall.	0 if vitamin A dose not received;
		1 if vitamin A dose received
Child deworming treatment	Proportion of children aged 6 to 23 months who received deworming treatment in the 6 months previous to the survey. Based on caregiver’s recall.	0 if deworming treatment not received;
		1 if deworming treatment received
Maternal level		
Maternal malnutrition	Undernutrition defined as mid-upper arm circumference (MUAC) below 210 mm for non PLW; below 230 mm for PLW [26].	0 not malnourished;
		1 malnourished
Hand-washing knowledge	Defined as good if three or more occasions when it is important to wash hands were given; satisfactory if two occasions were given; poor if one or none of the options were given.	2 good
		1 satisfactory
		0 poor
Health-seeking behaviour	Proportion of caregivers who sought medical care in case of illness (diarrhoea, fever or cough) of the child.	0 medical care was not sought;
		1 medical care was sought
Workload during pregnancy	Perceived workload during pregnancy compared with the time before the pregnancy. Possible answers: ‘less’, ‘more’ or ‘the same.	Less
		More
		The same
Food habits during pregnancy	Changes in the amount and types of food eaten during pregnancy compared with the time before pregnancy. Based on caregiver’s recall. Possible answers: ‘eaten less’/‘fewer types’; ‘eaten the same amount’/‘the same types’; ‘eaten more’/‘more food types’.	Less amount/fewer types;
		Same amount/same number of types;
		Bigger amount/more types.
Vitamin A supplementation during pregnancy	Proportion of mothers who received vitamin A dose (capsule) in the first two months after delivery	0 not received;
		1 received
Iron supplementation during pregnancy	Proportion of mothers who received any iron table or iron syrup during their last pregnancy	0 not received;
		1 received
Advice on child and maternal nutrition	Proportion of caregivers who ever received information on maternal and child nutrition by health workers (hospital or health facility or feeding centre staff, midwives or village health workers), or traditional/religious leaders, or other relatives and friends. Based on the caregiver’s recall.	0 never received;
		1 received
Decision-making to feed	Who in the family makes the decision regarding what to feed the children.	1 mother;
		2 others
Household level		
Household dietary diversity score [27]	Number of food groups consumed by the household’s member in the 24 h before the survey. Categorization defined by the authors, as no standard thresholds exist.	0–4
		5–8
		9–12
Farm production diversity score	Number of food groups produced by the household in the past 3 months. Categorization defined by the authors, as no standard thresholds exist.	0 (no production)
		1–2
		3–5
		6–12
Source of drinking water	Defined as improved if water coming from piped water, protected well, protected spring and bottled water. Otherwise = unimproved.	0 unimproved;
		1 improved
Hand-washing station	Defined as functional if a station was present, or water and soap or ash were available; non-functional if otherwise.	0 non-functional;
		1 functional
Use of iodized salt	Whether the household used iodized salt or not.	0 non-iodized salt;
		1 iodized salt

### Quality control

The SMART methodology was used to ensure standardized procedures and tools [28]. After a 6-day training, data collectors had to pass a standardization test to assess accuracy and precision of their anthropometric measurements. The questionnaire was piloted and finalized. Team leaders ensured data quality during data collection. Checks, skip functions and acceptable ranges were pre-established in the DDG devices to reduce mistakes. Implausible anthropometric measurements were defined as  ±6 SD, as per WHO criteria [29].

### Statistical analysis

The analysis of factors associated with stunting was divided by age groups: 6–23 and 24–59 months, and conducted for the entire sample and broken down by region. Baseline sociodemographic and clinical characteristics of the sample were described with simple frequency distribution. Crude associations between stunting and sociodemographic and clinical variables were investigated using Pearson’s chi-square test. A multivariate logistic regression model was constructed to identify factors associated with stunting. Odds ratios (OR), 95% confidence intervals (CI) and *p*-values were obtained. *P*-values  <0.05 were considered significant. Sampling weights were applied to ensure the representativeness of the sample at the regional level. The analysis was conducted in STATA IC/12.1 for Windows and SPSS 20.0. Anthropometric indicators were calculated with ENA software.

### Ethical considerations

Concern Worldwide routinely conducts nutrition surveys within its programmes, which are not subject to research ethical scrutiny. The organization subscribes to the ethical principles outlined in the Humanitarian Charter [30]. Furthermore, the project protocol and questionnaire were reviewed and approved by the Government of Tanzania and UNICEF. Oral informed consent was obtained by the interviewees. Consent to conduct anthropometric measurement was obtained from a parent or guardian in the local language.

## RESULTS

Data were collected on a total of 3280 children aged 6–59 months. The region or district was missing in 16 records that were excluded, leading to a sample of 3264 children. Descriptive statistics are presented in Table 2. We further excluded 17 records from the inferential analysis due to implausible anthropometric measurements. The final sample includes 3247 children aged 6–59 months, of which 1360 are in the age group of 6–23 months.

**Table 2.** Distribution of children aged 6–23 and 24–59 months by demographic, maternal and household characteristics, Iringa, Njombe and Mbeya regions, Tanzania, 2013

Variables	6–23 months		24–59 months	
	*n*	% or * Mean (SD)	*n*	% or * Mean (SD)
Nutritional status				
Stunting	1352	42.2	1895	45.7
Height for age		−1.79 (1.31)		−1.99 (1.21)
Child’s age (in months)	1360	14.2 (5.2)*	1904	38.4 (10.3)*
6–11		34.5		
12–23		65.5		
24–35				43.2
36–47				32.4
48–59				24.4
Child’s sex	1360		1904	
Female		47.6		50.3
Male		52.4		49.7
Child living with	1338			
Both parents		83.1		
Mother only		14.4		
Other guardians		2.5		
Minimum diet diversity (MAD)	1360			
No		84.8		
Yes		15.2		
Minimum meal frequency (MMF)	1336			
No		73.2		
Yes		26.8		
Minimum adequate diet (MAD)	1337			
No		99.2		
Yes		0.8		
Child’s consumption of animal source food	1338			
At least one food		32.1		
No		67.9		
Child’s consumption of vitamin-A-rich food	1337			
Yes		13.8		
No		86.2		
Child’s consumption of pulse/legumes/nuts	1337			
Yes		37.2		
No		62.8		
Snack between the meals	1330			
No snack		80.4		
One snack		12.0		
Two snacks		7.6		
Child morbidity	1360			
No illness		55.7		
One illness		23.4		
Two or three illnesses		20.9		
Health care-seeking behaviour in case of illness	550			
Care sought		71.6		
Care not sought		28.4		
Child received deworming treatment	1281			
Yes		51.1		
No		48.9		
Child received vitamin A supplementation	1285			
Yes		80.8		
No		19.2		
Maternal MUAC (in cm)	1309	27.8 (3.3)*	1694	28.1 (3.4)*
Malnourished		2.1		0.9
Non-malnourished		97.9		99.1
Mother currently pregnant	1348		1856	
Yes		2.9		10.2
No		97.1		89.8
Mother currently breastfeeding	1360		1904	
Yes		74.0		92.3
No		24.0		7.7
Birth in the past 5 years	1357		1902	
1 child		77.3		85.2
2+ children		22.7		14.8
Maternal hand-washing knowledge	1357		1902	
Poor		16.2		17.8
Satisfactory		44.1		51.9
Good		39.7		30.3
Hours the mother is away from home	1360		1904	
No		46.4		42.6
1–3 h		21.2		20.5
4–7 h		21.8		22.4
8–24 h		10.5		14.6
Husband support during pregnancy	1019			
Yes		73.1		
No		26.9		
Amount of food during pregnancy	1009			
Less		45.1		
The same		39.1		
More		15.8		
Types of food during pregnancy	1011			
Fewer		37.2		
The same		45.2		
More		17.6		
Mother received iron during pregnancy	1004			
Yes		62.0		
No		38.0		
Mother received vitamin A dose after delivery	990			
Yes		41.8		
No		58.2		
Advice on child and maternal nutrition	1054			
Yes		41.9		
No		58.1		
Decision-making to feed	1063			
Mother		75.0		
Others		25.0		
Source of drinking water	1357		1902	
Improved		68.4		70.7
Not improved		31.6		29.3
Hand-washing station	1360		1904	
Functional		5.6		7.9
No or not functional		94.4		92.1
Household dietary diversity score	1353	5 (4–6)*^,2^	1894	5 (4–6)*^,^^a^
1–4		40.7		36.7
5–8		56.5		59.5
9–12		2.8		3.8
Household farm production diversity score	1349	3 (1–4)*^,2^	1886	3 (2–5)*^,^^a^
No		13.7		11.6
1–2		30.3		24.1
3–5		40.8		42.3
6–12		15.1		22
Home/kitchen garden	1360		1904	
Yes		26.6		26.6
No		73.4		73.4
Frequency of buying fresh food	1357		1902	
Daily		44.6		47.9
2–3 times/week		34.7		30.7
Once a week		10.6		11.1
Less often		10.1		10.2
Production of animal source food	1360		1904	
At least one food		60.5		65.6
No		39.5		34.4
Consumption of animal source food	1360		1904	
At least one food		51.0		48.8
No		49.0		51.2
Production of vitamin A-rich food	1360		1904	
At least one food		38.8		47.2
No		61.2		52.8
Consumption of vitamin A-rich food	1360		1904	
At least one food		80.2		83.5
No		19.8		16.5
Use of iodized salt	1332		1871	
Yes		79.3		79.2
No		20.7		20.8
Place of residence	1360		1904	
Urban		18.7		20.5
Rural		81.3		79.5
Region	1360		1904	
Mbeya		63.8		67.4
Njombe		14.8		13.4
Iringa		21.4		19.2

^a^Median and interquartile range.

Mean HAZ was −1.79 among 6–23-month-old children and −1.99 in the older group. Prevalence of stunting was 42.2%, and 45.7%, respectively.

Table 3 presents results from the bivariate and multivariate logistic regression for 6–23-month-old children, and Table 4 for the age group 24–59 months. In both groups and after adjusting for confounding factors, children in Njombe and Iringa have higher odds of being stunted than children in Mbeya. Across regions, children in their second and third year of life have higher odds to be stunted than younger or older children. Male children have higher odds than female.

**Table 3.** Crude and adjusted odds ratio (95% CI) for stunting among children aged 6 to 23 months, overall and disaggregated by region, according to child, maternal and household characteristics, southern highlands, Tanzania, 2013 (only significant results are shown)

Variables	Overall (*N* = 929)		Mbeya (*N* = 374)		Njombe (*N* = 348)		Iringa (*N* = 357)	
	Crude OR	AOR	Crude OR	AOR	Crude OR	AOR	Crude OR	AOR
Male sex	2.15***	2.17***	2.35***	2.90***	1.67***	2.13***	2.04***	2.45***
	(1.65–2.81)	(1.52–3.09)	(1.57–3.50)	(1.76–4.80)	(1.15–2.42)	(1.27–3.60)	(1.41–2.95)	(1.44–4.16)
Child age								
6–11	1	1	1	1	1	1	1	1
12–24	2.70***	3.91***	2.730***	4.63***	2.92***	3.04***	2.63***	4.74***
	(2.02–3.62)	(2.49–6.12)	(1.751–4.256)	(2.47–8.67)	(1.97–4.34)	(1.68–5.50)	(1.75–3.95)	(2.40–9.37)
Receiving deworming treatment		0.64**		0.53**			1.89***	
		(0.41–0.99)		(0.30–0.93)			(1.28–2.79)	
Mother hours away from home								
0 h	1	1	1	1				
1–3 h	0.85	1.00	0.79	0.96				
	(0.59–1.21)	(0.64–1.59)	(0.48–1.32)	(0.53–1.76)				
4–7 h	1.64***	1.93***	1.90**	2.25**				
	(1.17–2.30)	(1.21–3.07)	(1.14–3.14)	(1.20–4.19)				
8–24 h	0.83	1.07	0.79	1.00				
	(0.53–1.29)	(0.58–1.98)	(0.39–1.60)	(0.42–2.39)				
Mother currently pregnant					2.98**	4.26**		6.64**
					(1.05–8.51)	(1.08–16.87)		(1.23–35.66)
Food types during last pregnancy								
Fewer							1	
The same							1.79**	
							(1.13–2.84)	
More							1.10	
							(0.64–1.88)	
Receiving husband support during pregnancy					0.61**			
					(0.38–0.99)			
Births in the past 5 years								
One								1
Two or more								2.02**
								(1.11–3.66)
Household dietary diversity score								
0–4	1	1	1	1			1	
5–8	0.66***	0.61**	0.66**	0.63			0.61***	
	(0.50–0.86)	(0.41–0.92)	(0.44–0.98)	(0.36–1.09)			(0.42–0.89)	
9–12	0.25***	0.15***	0.20**	0.14**			0.10**	
	(0.09–0.66)	(0.04–0.53)	(0.04–0.95)	(0.02–0.96)			(0.01–0.82)	
Owning a home garden		0.58**						
		(0.38–0.89)						
Production of animal-based food					0.67**	0.50**		
					(0.46–0.98)	(0.26–0.95)		
Production of vitamin A-rich fruits and vegetables	1.43***	1.71**			1.59**			
	(1.09–1.87)	(1.06–2.76)			(1.09–2.32)			
Frequency of buying fresh food								
Daily					1			1
2–3 times per week					1.20			2.19**
					(0.78–1.85)			(1.14–4.23)
Once a week					1.15			1.47
					(0.66–2.00)			(0.53–4.05)
Less often					2.28**			1.11
					(1.19–4.39)			(0.41–2.99)
Using iodized salt	0.72**						0.54***	0.41***
	(0.53–0.97)						(0.34–0.84)	(0.21–0.79)
Knowledge of hand-washing practices								
Poor								1
Satisfactory								3.10***
								(1.33–7.24)
Good								1.13
								(0.47–2.71)
Improved water source							0.48***	
							(0.30–0.76)	
Urban residence	0.68**						0.50***	
	(0.49–0.95)						(0.30–0.81)	
Region								
Mbeya	1	1						
Njombe	1.34**	1.77**						
	(1.03–1.76)	(1.11–2.80)						
Iringa	1.19	1.59**						
	(0.91–1.55)	(1.06–2.40)						

Level of significance:. ***p  <  0.01,**p  <  0.05; OR =  Odds ratio.

**Table 4.** Crude and adjusted odds ratio (95% CI) for stunting among children aged 24 to 59 months, overall and disaggregated by region, according to child, maternal and household characteristics, southern highlands, Tanzania, 2013 (only significant results are shown)

Variables	Overall (*N* = 1618)		Mbeya (*N* = 561)		Njombe (*N* = 517)		Iringa (*N* = 535)	
	Crude OR	AOR	Crude OR	AOR	Crude OR	AOR	Crude OR	AOR
Child age								
24–35	1	1	1	1	1	1		
36-47	0.73**	0.71**	0.70**	0.69*	0.68**	0.68*		
	(0.57–0.94)	(0.54–0.95)	(0.49–1.00)	(0.45–1.05)	(0.47–0.99)	(0.44–1.06)		
48-59	0.59***	0.60***	0.52***	0.53***	0.64**	0.61**		
	(0.44–0.78)	(0.44–0.83)	(0.35–0.77)	(0.33–0.85)	(0.43–0.97)	(0.37–0.98)		
Male sex	1.30**	1.28**						
	(1.04–1.61)	(1.00–1.64)						
Mother currently breastfeeding	1.79***	1.97***		2.82**				
	(1.20–2.68)	(1.18–3.29)		(1.23–6.47)				
Mother currently pregnant					2.28**	2.81**		
					(1.05–4.93)	(1.20–6.60)		
Mother hours away from home								
0 h				1	1	1	1	
1–3 h				0.58**	0.98	1.20	1.32	
				(0.36–0.94)	(0.63–1.53)	(0.72–2.00)	(0.86–2.01)	
4–7 h				0.81	1.64**	1.66**	1.63**	
				(0.50–1.30)	(1.07–2.52)	(1.01–2.73)	(1.09–2.44)	
8–24 h				0.97	1.53*	1.95**	1.31	
				(0.55–1.71)	(0.97–2.39)	(1.15–3.31)	(0.76–2.27)	
Improved water source		0.70**		0.58***	1.56**			0.63**
		(0.52–0.93)		(0.39–0.87)	(1.06–2.29)			(0.40–0.99)
Functional hand-washing station		0.63**		0.43**				
		(0.40–0.98)		(0.18–0.99)				
Knowledge of hand-washing practices								
Poor							1	1
Satisfactory							0.81	0.80
							(0.51–1.28)	(0.47–1.36)
Good							0.55**	0.47**
							(0.34–0.90)	(0.26–0.85)
Household dietary diversity score								
0–4	1		1					
5–8	0.66***		0.65**					
	(0.53–0.83)		(0.47–0.91)					
9–12	0.96		1.25					
	(0.52–1.80)		(0.59–2.66)					
Household consuming animal-based food	0.67***		0.63***	0.63**				
	(0.54–0.84)		(0.46–0.86)	(0.42–0.94)				
Farm production diversity score								
No production	1						1	
1–2 food groups	0.90						1.79**	
	(0.67–1.20)						(1.01–3.14)	
3-5 food groups	1.56**						2.33***	
	(1.04–2.33)						(1.38–3.93)	
6–12 food groups	1.34						3.16***	
	(0.92–1.94)						(1.72–5.78)	
Household production of animal-based food			0.72**				1.45**	
			(0.52–0.99)				(1.04–2.02)	
Production of vitamin A-rich fruits and vegetables							2.25***	
							(1.63–3.10)	
Owning a home garden							1.99***	
							(1.44–2.75)	
Frequency of buying fresh food								
Daily							1	
2–3 times per week							1.46**	
							(1.00–2.11)	
Once a week							1.64*	
							(0.99–2.72)	
Less often							1.30	
							(0.80–2.13)	
Urban residence			1.74***	2.02***		0.61**	0.47***	
			(1.18 – 2.57)	(1.21 – 3.39)		(0.39 – 0.95)	(0.31 – 0.72)	
Region								
Mbeya								
Njombe	1.83***	1.77***						
	(1.47–2.29)	(1.33–2.35)						
Iringa	1.27**	1.37**						
	(1.02–1.59)	(1.04–1.79)						

*Note*. OR =  Odds ratio.

Level of significance:

***p  <  0.01,

**p  <  0.05.

In the entire sample, children aged 6–23 months had lower odds of stunting if they received deworming treatment or came from a household consuming five or more food groups or that has a home garden. In Njombe, children from households producing animal-based food have lower odds of stunting. In Iringa, using iodized salt was also found to be protective. Risk factors in the same age group are maternal absence for 4–7 h and production of vitamin-A-rich food. In Njombe and Iringa, children whose mother is currently pregnant have higher odds of stunting. In Iringa, two or more births in the past 5 years, reduced frequency of buying fresh vegetables and improved knowledge of hand-washing practices were other risk factors.

Overall, children aged 24–59 months from households with access to improved water source or with a functioning water station had lower odds of stunting. Knowledge of hand-washing practices protects against stunting in Iringa, while in Mbeya, children from households consuming animal-based food have lower odds. In the entire sample, children whose mother was currently breastfeeding have higher odds of stunting. Short maternal absence (1–3 h) was a protective factor in Mbeya, while longer absence (4–7 h) was a risk factor in Njombe. Urban residence was a protective factor in Njombe and a risk factor in Mbeya.

## DISCUSSION

This study investigates factors associated to the nutritional status of pre-school children in Tanzanian southern highlands. The likelihood of stunting is higher among children in the regions of Njombe and Iringa, compared with Mbeya. As the regions have similar socioeconomic profiles, further effort is needed to investigate possible explanatory factors to better define interventions. For example, diet habits, cultural practices and HIV prevalence could play a role. Children under 2 years of age consume more dairy products in Mbeya than in the other regions (results not shown), which is associated with improved linear growth [31]. The higher HIV/AIDS prevalence in Njombe (15%) compared with Iringa and Mbeya (9%) [32] can also have implications on the households’ economic status and indirectly on the child nutritional status [33]: reduced labour productivity, increased expenditure in illness and funerals and reduced food security. Njombe registers, for example, the lowest mean household dietary diversity score (HDDS), followed by Iringa and Mbeya (difference among regions is significant). Within Mbeya though, urban children seem to be more disadvantaged than their rural counterparts, possibly due to the fast urbanization that was not followed by an equitable development of and access to health, sanitation and educational services [34].

With regard to child characteristics, our analysis confirms that children in their second and third year of life are more likely to be stunted than both younger and older children. This is well-recognized in the literature. Studies have shown that children accumulate growth delay during the first 2 years of life, with stunting peaking around 2 and 3 years, after which they stabilize [12, 35]. Furthermore, boys are at higher risk than girls, which also confirms results from sub-Saharan African and Asian countries [36].

The nutritional status of a child is directly related to maternal presence and her reproductive status. Maternal time allocation affects both the child nutritional status (through the time spent caring for the child) and income generation (through labour force) [37]. The net effect may vary by household and by child age. Our study shows that small children are particularly affected by maternal absence, while the results are mixed among older children. Furthermore, in Njombe and Iringa, children of pregnant women and of women with recent short birth intervals are more likely to be stunted. Among the older children, those whose mother is breastfeeding have higher odds. These results point in the same direction: older children may suffer from the presence of younger siblings. Programmes supporting mothers during pregnancy and lactation can have positive effects not only on the newborn, but also on older siblings. Cost–benefit studies of such interventions should also take this into account. Various activities could contribute towards reducing the workload on women: conservation agriculture, shortening distance to drinking water sources and engaging with other family members. Reproductive health programmes involving men and boys represent an important channel to emphasize men’s shared responsibility in pregnancy and childcare [38]. A better understanding of cultural norms defining women’s and men’s reproductive role in the Tanzanian southern highlands is necessary to design successful programmes. Finally, our study supports the evidence on promoting child spacing and family planning, which can reduce maternal burden and has been shown to contribute to a reduction of stunting [39].

It is widely accepted that economic welfare boosts nutritional status. Studies from resource-limited settings show that children from families with greater income and resources tend to have better diets, improved nutritional status and an overall growth-conducive environment [16, 40]. Our study contributes to this evidence by showing that higher HDDS is associated with lower odds for stunting among 6–23-month-old children. The number of food groups consumed in a household is commonly used as a proxy for the socioeconomic level, as it reflects the economic access to a variety of foods [27]. The interpretation of the relation between HDDS and stunting is complicated by the fact that richer families usually have better access to health care and improved environmental health, therefore pointing to a broader relation between poverty and stunting. Cultural food practices may have a strong mediating influence on a child nutrition status regardless of the family economic conditions. Further operational research to unravel the pathways should be undertaken.

Access to improved water source and to a functioning hand-washing station protects against stunting, particularly among older children. These two public health interventions are likely to reduce the transmission of diarrhoeal diseases and the risk of tropical enteropathy [41], which are both associated with reduced linear growth. As 43% of the Tanzania households still do not have access to improved water source [21], strengthening strategies to increase the provision of water and sanitation interventions is crucial for the health of preschool children.

Surprisingly, the production of vitamin-A-rich food was found to increase the odds of stunting, instead of reducing it. It should be investigated further whether producing vitamin-A-rich food substitutes the production (and consumption) of animal-based food, which may have a greater impact on child growth.

None of the IYCF indicators was associated with linear growth, despite evidence from multi-country studies that indicate that diverse [42, 43] and adequate diet, as well as solid food consumption [44], reduce the risk of stunting. In the same studies, no association is found between meal frequency and linear growth, as in our analysis. Very few children (<1%) in our study met the definition of adequate diet, unlike the National Nutrition Survey, where 7.3%, 5.3% and 23.9% of the children in Iringa, Mbeya and Njombe, respectively, received minimum adequate diet (MAD). The results are not comparable unfortunately, as the calculation of MAD in the National Nutrition Survey did not consider milk feeds for non-breastfed children as recommended by the WHO.

The present study has some limitations. First, the cross-sectional design does not allow to investigate causation, but only association. Second, information on maternal education was not collected, despite the importance recognized to it in the literature. The SMART questionnaire should include it in the future. Third, it was not possible to investigate the extent to which HIV/AIDS influences stunting in this population. Given the complexity and sensitivity around HIV status, data on HIV prevalence were not collected.

In conclusion, our analysis confirms that stunting remains a public health problem in the southern highlands region in Tanzania. Interventions aiming to improve household wealth and sanitation conditions to reduce the high level of stunting should be put in place. Furthermore, family planning activities as well as supporting programmes for mothers during pregnancy and lactation can have positive effects not only on the newborn, but also on older siblings.
